# Assessing the Efficacy of Nano- and Micro-Sized Magnetic Particles as Contrast Agents for MRI Cell Tracking

**DOI:** 10.1371/journal.pone.0100259

**Published:** 2014-06-24

**Authors:** Arthur Taylor, Anne Herrmann, Diana Moss, Violaine Sée, Karen Davies, Steve R. Williams, Patricia Murray

**Affiliations:** 1 Institute of Translational Medicine, University of Liverpool, Liverpool, United Kingdom; 2 Institute of Integrative Biology, University of Liverpool, Liverpool, United Kingdom; 3 Centre for Imaging Sciences, University of Manchester, Manchester, United Kingdom; Institute for Frontier Medical Sciences, Kyoto University, Japan

## Abstract

Iron-oxide based contrast agents play an important role in magnetic resonance imaging (MRI) of labelled cells *in vivo*. Currently, a wide range of such contrast agents is available with sizes varying from several nanometers up to a few micrometers and consisting of single or multiple magnetic cores. Here, we evaluate the effectiveness of these different particles for labelling and imaging stem cells, using a mouse mesenchymal stem cell line to investigate intracellular uptake, retention and processing of nano- and microsized contrast agents. The effect of intracellular confinement on transverse relaxivity was measured by MRI at 7 T and in compliance with the principles of the ‘3Rs’, the suitability of the contrast agents for MR-based cell tracking *in vivo* was tested using a chick embryo model. We show that for all particles tested, relaxivity was markedly reduced following cellular internalisation, indicating that contrast agent relaxivity in colloidal suspension does not accurately predict performance in MR-based cell tracking studies. Using a bimodal imaging approach comprising fluorescence and MRI, we demonstrate that labelled MSC remain viable following *in vivo* transplantation and can be tracked effectively using MRI. Importantly, our data suggest that larger particles might confer advantages for longer-term imaging.

## Introduction

Monitoring the migration and fate of cells *in vivo* plays an important role in the development of cellular therapies. In such therapies, where cells are administered to an animal or patient in order to treat a disease, there is a need to image the therapeutic cells non-invasively in order to assess their delivery, migration over time and viability. Currently, a wide range of techniques based on optical, photoacoustic, nuclear and magnetic resonance imaging (MRI) are available for the pre-clinical tracking of cells[1], [2]. In all of these methods, the therapeutic cells need to be labelled prior to imaging in order to allow them to be distinguished from the host cells. This is normally achieved with the introduction of contrast agents (such as quantum dots, gold nanoparticles and radioactive tracers)[1], [2] or reporter genes (such as fluorescent proteins, luciferase and HSV1-tk)[2], [3] into the cells. In the clinic, the method of choice for cellular imaging *in vivo* has been MRI as it is safe and does not suffer from limitations associated with other techniques, such as low penetration depth (*e.g.* fluorescence and bioluminescence imaging) or poor spatial resolution (*e.g.* nuclear imaging). Additionally, until recently, the commercial availability of MR contrast agents clinically approved by regulatory agencies provided a means to label cells for administration in humans[4], [5], [6], [7]. Although such contrast agents were not marketed for this purpose, as they were designed to be liver-specific contrast agents[8], they were successfully used *off label* for cell tracking. For this application, the cells of interest are labelled *in vitro* prior to their administration with uptake being achieved either via spontaneous endocytosis when the contrast agent is added to the culture medium or with use of transfection aids[1].

Such MRI contrast agents are based on superparamagnetic iron oxide nanoparticles (SPION). When cells labelled with SPION are introduced into an animal, the magnetic nanoparticles within the cells locally disturb the magnetic field homogeneity[8] during MR imaging, thus allowing them to be distinguished from the tissue background in T_2_ and T_2_*-weighed images. Since the first reports that introduced the concept of MR cell imaging using SPION in the early 1990s[9], numerous research groups have used this method in pre-clinical and clinical studies.

The ready availability of commercial SPION, as well as the well-established procedures for synthesising such nanoparticles[10] has facilitated the widespread application of this technique for cell tracking. However, most studies on cell tracking tend to focus on the use of a particular type of SPION, or on methods to enhance cell labelling, and only a few actually compare the performance of different types of SPION *in vitro* and *in vivo*. In this work, three commercially available iron oxide-based contrast agents with distinct physical properties (size, number of magnetic cores, surface chemistry and, consequently, T_2_ relaxation rates) are evaluated as a means to label and image mesenchymal stem cells (MSC) using MRI. Our goals were to establish their uptake levels in MSC, the intracellular fate immediately after labelling and during cell proliferation, as well as their imaging properties *in vitro* and *in vivo*. Moreover, an important aim of our work was to establish an test system *in vivo* that complied with the principles of the ‘3Rs’ (i.e. Replacement, Refinement, Reduction in research using animals), for according to 3Rs philosophy, it would be deemed inappropriate to use protected animals such as laboratory rodents for evaluating the effectiveness of tracking agents if alternative models were available. For this reason, we have used the chick embryo as a test bed for evaluating the different contrast agents, for during the initial stages of development, the chick embryo is not considered to be sentient, and furthermore, unlike the situation with mammalian embryos, the mother does not need to be sacrificed in order to gain access to the embryo. Although all products evaluated in this work were found to be suitable for cell labelling and imaging *in vivo*, marked differences were seen in labelling efficiency, the extent of dilution upon cell division, the extent of dissolution in a lysosomal microenvironment model and also, on T_2_ relaxation as measured in solution or within cells. These effects are discussed in regard to the application of iron oxide-based particles as contrast agents for cell tracking using T_2_-weighed images.

## Methods

### Contrast Agents and Cell Labelling

**Figure 1:**
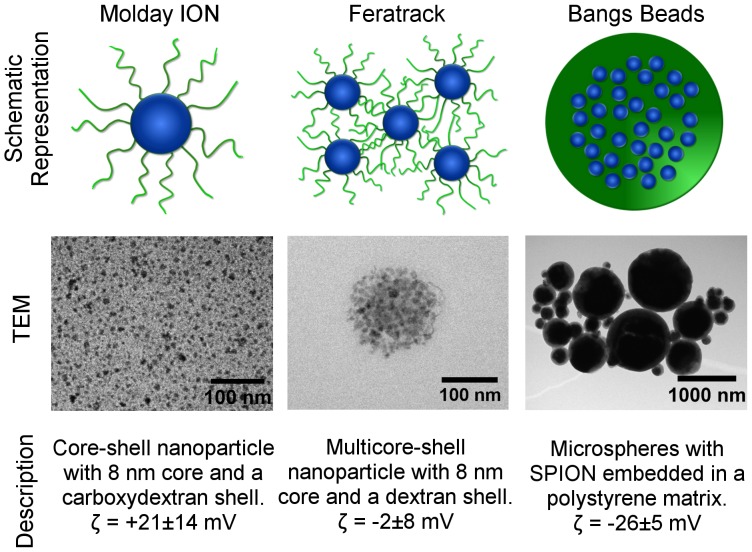
Properties of contrast agents. Schematic representation, transmission electron microscopy images and description of the contrast agents used in this study. Zeta potential (ζ) was assessed in 10 mM NaCl at a concentration of 100 µg[Fe]/ml.

Molday ION Evergreen (Biopal Inc, Worcester, MA, USA), Feratrack (Miltenyi Biotec, Surrey, England) and Dragon Green Encapsulated Magnetic Polymers (Bangs Beads, Stratech Scientific, Suffolk, England) were used for cell labelling. Zeta potential was measured in 10 mM NaCl using a Zetasizer Nano Z (Malvern, England) and averaged over 50 runs. A schematic representation of the contrast agents, including a description of their properties is shown in Figure 1.

The multipotent MSC line D1 ORL UVA (ATCC CRL-12424) was used as a model and grown in Dulbecco's Modified Eagle's Medium (DMEM) containing 10% fetal calf serum (FCS) at 37°C under a humidified atmosphere with 5% CO_2_. For labelling with the magnetic particles, 5×10^4^ cells were seeded in 24-well plates and allowed to attach and grow for 24 h. After this period the cells reached approximately 50% confluency. The culture medium was then replaced with 350 µl of fresh medium containing the contrast agents and the cells were allowed to grow for a further 24 h. In all cases the contrast agents were added directly to the complete culture medium with the exception for Feratrack, where labelling was also performed in accordance with manufacturer's instructions, which involves the use of a lipid-based loading reagent in conjunction with serum free medium; with this reagent, cells were labelled for 5 h followed by a recovery period of 19 h. After the labelling period, the cells were carefully washed with phosphate-buffered saline (PBS) to remove excess contrast agent and used for downstream studies.

### Quantification of Intracellular Iron & Labelling Efficiency

Intracellular iron quantification was carried out using a slightly modified version of a previously reported colourimetric method[11]. Briefly, 5×10^4^ cells were suspended in 100 µl acetone and digested for at least 2 h after which an equal volume of 1.2 M hydrochloric acid was added to the sample that was then heated at 75°C for 2 h. After this treatment, the samples were allowed to cool down and 60 µl of ferrozine reagent (6.5 mM Ferrozine, 13.1 mM neocuproine, 2 M L-ascorbic acid and 5 M ammonium acetate, all from Sigma, Dorset, England) was added to the sample. The absorbance was measured at 570 nm and compared to a standard curve prepared with an iron standard (TraceCERT, Sigma). Flow cytometry was used to assess the labelling efficiency (percentage of labelled cells) and also as a qualitative means to evaluate intracellular concentration of contrast agents. Cells were fixed with 4% formaldehyde and the green fluorescence intensity of at least 1×10^4^ cells was measured with a FACScalibur flow cytometer (BD Biosciences, Oxford, England). As Feratrack is not conjugated to a fluorophore, an anti-dextran FITC-conjugated antibody (Stem Cell Technologies, Grenoble, France) was used to detect the intracellular presence of this contrast agent. For that purpose, cells were permeabilised with 0.1% saponin (Sigma), blocked with 10% bovine serum albumin and allowed to react with the antibody for 2 h in the dark using concentrations suggested by the manufacturer.

### Cellular Imaging

Fluorescence microscopy was used to identify the cellular localisation of the contrast agents. Cells were grown and labelled in culture slides (BD Biosciences) and the same immunostaining procedures as described for flow cytometry were applied. For imaging the lysosomes, a rabbit antibody against the Lysosomal-Associated Membrane Protein 2 (LAMP2, Abcam, Cambridge, England) was used in conjunction with secondary goat anti-rabbit antibody conjugated to Alexa-Fluor-488 (Life Technologies, Paisley, Scotland). Particles and lysosomes were imaged using confocal microscopy (Leica TCS SP II) using a pinhole size of 1 airy unit. Nuclei were stained using 4′,6-diamidino-2-phenylindole (DAPI, Life Technologies) and imaged using wide-field fluorescence.

For transmission electron microscopy (TEM) cells were grown and labelled in 3.5 cm dishes, followed by fixation with 4% formaldehyde/2.5% glutaraldehyde, post fixation with 1% osmium tetroxide, dehydration and embedding in epoxy resin. Thin sections (70 nm) were then collected over copper grids containing a formvar support film. For analysis of the contrast agents, those were simply dispersed of over the grids containing the support film and allowed to dry. Samples were analysed with a FEI Technai G2 Spirit BioTwin microscope operated at 100 kV.

### Intracellular Retention and Stability of Contrast Agents

To quantify the intracellular retention of contrast agents and the effect on cellular proliferation, labelled cells were trypsinised after labelling and counted using an automated cell counter (TC20, Biorad). A fraction of the cells (5×10^4^) was plated in 24-well plates in triplicate and allowed to grow for up to 3 days. At 24 h intervals, the cells from one of the wells were trypsinised, counted using the trypan blue exclusion assay and then used for intracellular iron quantification.

To evaluate the long-term stability of the particles, a lysosomal model of cellular digestion as originally proposed by Skotland *et al.* [12] was used. The pH of PBS containing 22 mM sodium citrate tribasic (Sigma) was left at 7.2 or adjusted to 5.5 or 4.5 using hydrochloric acid. A sample containing 1 µg of particles (Fe basis) was added to these buffers and allowed to digest for a period of up to 28 days at 37°C. The amount of dissolved iron was quantified using the ferrozine assay as described above using a slightly modified ferrozine reagent consisting of 6.5 mM Ferrozine, 100 mM L-ascorbic acid and 1.1 M ammonium acetate.

### Differentiation

The multipotent MSC D1 cell line was differentiated into adipocytes and osteocytes. For that purpose, the medium was exchanged immediately after labelling and replaced with fresh medium containing adipogenic or osteogenic supplementation. Adipogenic supplementation consisted of 100 nM Dexamethasone, 10 mM β-glycerophosphate disodium salt hydrate and 77 µM 2-Phospho-L-ascorbic acid trisodium salt (all from Sigma), whereas the osteogenic supplementation consisted of 100 nM Dexamethasone, 155 µM 2-phospho-L-ascorbic acid trisodium salt, 50 µM indomethacin and 175 nM bovine pancreas insulin (all from Sigma). The cells were then allowed to grow for a further 9 d with periodic medium changes. Effective differentiation was evaluated by fixation and histochemical staining with 0.5% Oil Red O (Sigma) in isopropanol or 2% Alizarin Red S (Sigma).

### Optical and Magnetic Resonance Imaging

All magnetic resonance data were acquired with a Bruker 7 T Avance III instrument using a 38 mm transmit/receive quadrature volume coil. To obtain the longitudinal relaxivity of the contrast agents, T_1_/T_2_ maps were generated using a modified Rapid Acquisition with Refocused Echoes (RARE) sequence with variable repetition times (TR) of 5000, 3000, 1500, 800, 400 and 200 ms, and echo times (TE) of 11, 22, 55, 77 and 99 ms. Paravision 5.0 (Bruker Biopsin) was used to determine the mono-exponential decay in signal intensity as a function of echo time and the respective relaxation time constant T_2_, which was then used to compute the relaxation rate R_2_ (taken as the reciprocal of the relaxation time). The relaxation rate of agarose without any contrast agent was used to normalise the data and obtain ΔR_2_. The same procedure was used to determine the relaxivity in cells, whereby the iron content per cell was calculated and used to determine the number of cells necessary to achieve the concentrations used for measurements. In all cases, particles and cells were suspended in 1% low-melting temperature agarose (Sigma) to prevent samples being subjected to temperatures above 40°C and then loaded into 200 µl polypropylene tubes which were held in place for axial imaging using a sample holder.

For imaging *in vivo* by fluorescence, cells were first transduced with viral particles encoding the dTomato gene. Viral particles were produced in HEK 293TN cells using three plasmids encoding the viral envelope (pMD2.G), packaging proteins (psPAX2) and the transfer vector (pHIV.dTomato), all obtained as gifts from Didier Trono and Bryan Welm (Addgene plasmids #12259, #12260 and #21374). The number of viral particles obtained was titred using HEK 293TN cells and the MSC were then transduced with a multiplicity of infection of 5 viral particles per cell yielding >90% of cells positive for dTomato 72 h post-transduction. No noticeable changes in the amount of contrast agent taken up were observed in relation to non-transduced cells. Those cells were then labelled with the magnetic particles as described. Fertilised white leghorn chicken eggs were incubated in a humidified incubator at 37°C. At embryonic day 3, eggs were windowed and 5×10^4^ labelled cells were implanted in the midbrain of the chick embryo *in ovo*. The chicks were allowed to grow up to embryonic day 5, at which point they were harvested from their eggs and imaged using a fluorescence stereoscope (Leica M165FC) before being fixed in 4% formaldehyde and embedded in 1% low-melting temperature agarose for MRI. Axial T_2_-weighed slices were acquired with a high resolution TurboRARE T_2_-weighed sequence with the following parameters: field of view 30×30 mm, matrix 256×256, slice thickness 0.5 mm, effective TE 33 ms, RARE factor 8, TR 2741.9 ms, averages 10, flip angle 135^o^, scan time 14 min 37 s).

## Results

### Uptake of contrast agents

The contrast agents evaluated consisted of SPION in the form of particles with a single core and a cationic carboxydextran shell (Molday ION), multicore particles and a dextran shell (Feratrack) or multicore microspheres embedded in a carboxyl-modified polystyrene matrix (Bangs Beads). The manufacturers' data indicates a hydrodynamic diameter of 35 nm for the single core particles, 60–140 nm for the multicore particles and an average size of 860 nm for the microspheres, the latter comprising a broad size distribution, in agreement with the TEM images obtained (Figure 1). Molday ION was found to be cationic with a surface charge of +21 mV (manufacturers' data suggests +31 mV), Feratrack neutral at −2 mV and Bangs Beads anionic at −26 mV.

**Figure 2:**
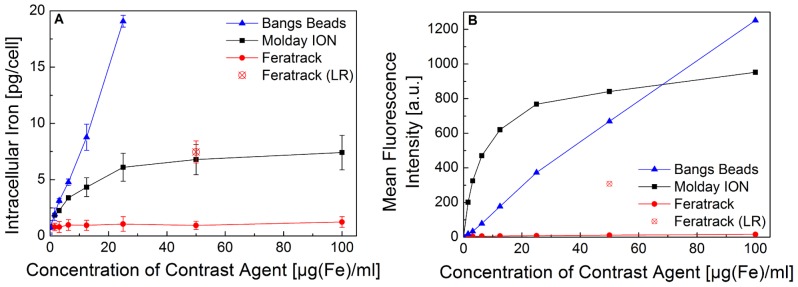
Intracellular iron content of murine mesenchymal stem cells as a function of labelling concentration. Cells (5×10^4^) were seeded in 24-well plates and labelled for 24 h by adding the contrast agents to the culture medium. Intracellular iron concentration was measured (A) quantitatively using the ferrozine assay (mean ± SD from three independent experiments) or (B) qualitatively using flow cytometry (representative data from three independent experiments). LR denotes labelling with loading reagent provided by the contrast agent manufacturer.

Uptake of the contrast agents was measured over a concentration range of 1.5–100 µg[Fe]/ml. Spontaneous uptake was obtained by simply adding the contrast agents to the media and was found to be concentration dependent for Molday ION and Bangs Beads, whereas virtually no uptake was seen for Feratrack (Figure 2A). The intracellular iron content after labelling cells with Molday ION was found to reach a plateau at about 25 µg[Fe]/ml, suggesting saturation of the cells. Uptake of Bangs Beads was linear up to a concentration of 25 µg[Fe]/ml. Given the size and density of these particles, they displayed a strong tendency to undergo sedimentation during the 24 h labelling period. This hindered an accurate evaluation of intracellular iron content at concentrations above this level, as extracellular aggregates were difficult to wash away, even with extensive PBS washes. Quantitative values are therefore not shown for these conditions. When labelling according to the manufacturer's instructions, which involves the use of a lipid-based loading reagent and an effective concentration of 50 µg[Fe]/ml, a significant intracellular uptake of Feratrack (Figure 2A) was observed, suggesting a stealth shell that requires the use of a transfection agent for effective labelling. To confirm the quantitative trends observed with the ferrozine assay, flow cytometry was carried out with the same cells. Molday ION and Bangs Beads are conjugated with a green fluorophore allowing the estimation of uptake by evaluation of the mean green fluorescence of individual cells. For Feratrack, an anti-dextran antibody conjugated to FITC was used in order to label the SPION. The trend in the mean fluorescence intensity as a function of contrast agent concentration was exactly the same as observed quantitatively by intracellular iron measurement (Figure 2B) suggesting saturation at ∼25 µg[Fe]/ml for Molday ION, the need of transfection aids for Feratrack and linear uptake for Bangs Beads. As flow cytometry allows the exclusion of extracellular aggregates by gating the data using forward and side scatter data plots, fluorescence intensity data are provided for concentrations above 25 µg[Fe]/ml suggesting that even higher amounts of Bangs Beads can be taken up by this cell line, although it was not possible to evaluate that quantitatively. For the purpose of comparing the efficacy and effects of the different contrast agents at similar intracellular concentrations, further studies were conducted with cells labelled with a defined set of standard labelling conditions summarised in Table 1. No significant statistical differences in intracellular concentrations between these conditions were found (p>0.05, 2-tailed, unpaired, student's t-test).

**Table 1:** Standard set of conditions used for labelling cells.

Contrast Agent	Labelling Concentration (µg[Fe]/ml)	Intracellular Concentration (pg[Fe]/cell)
**Molday ION**	25	6.09±1.23
**Feratrack**	50 (with LR)	7.46±0.98
**Bangs Beads**	12	8.77±1.16

LR: loading reagent, as provided by manufacturer.

### Intracellular localisation and stability

**Figure 3:**
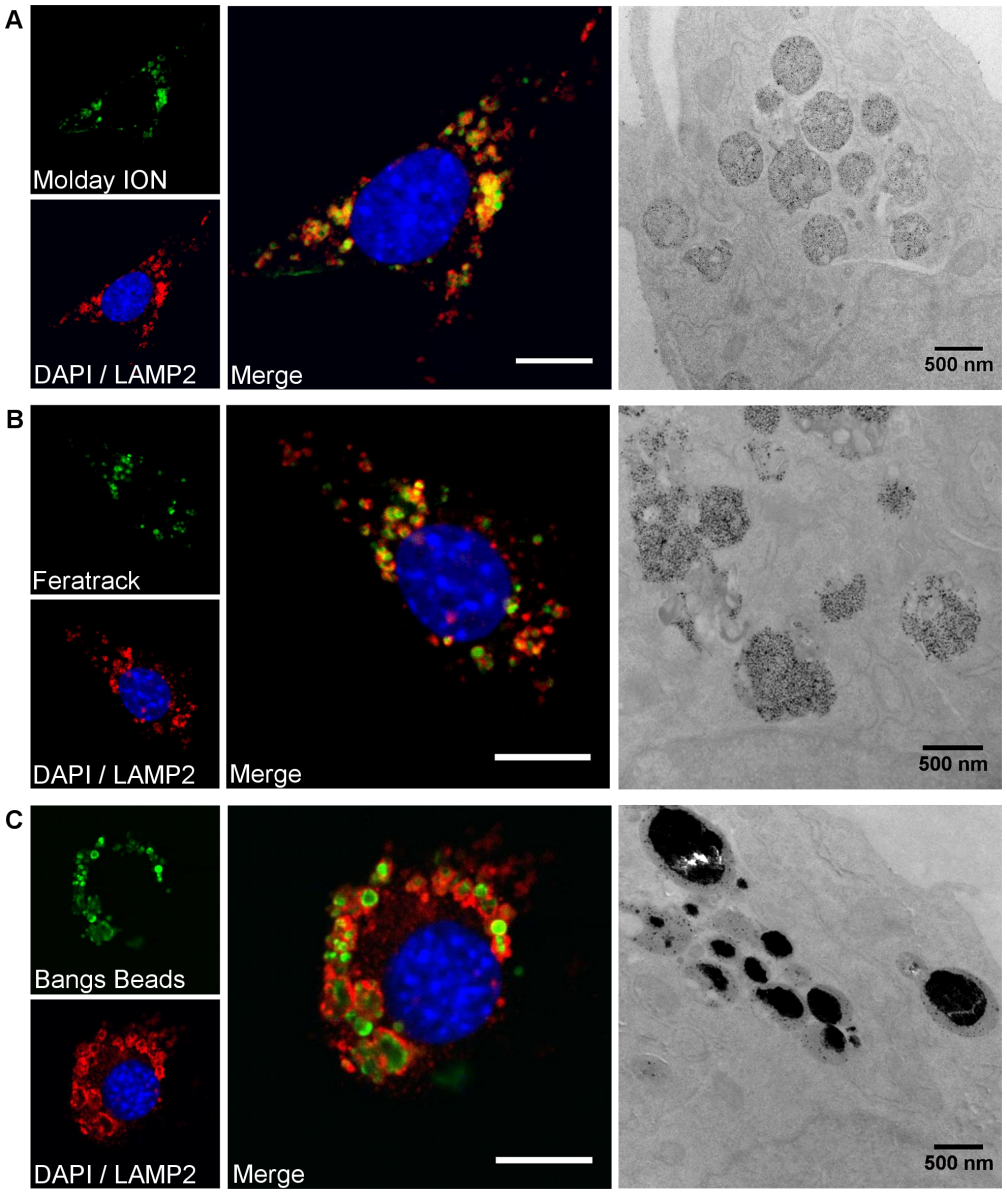
Confocal and electron microscopy images evidencing intracellular uptake and accumulation of contrast agents in lysosomes. Cells were labelled with (A) Molday ION, (B) Feratrack or (C) Bangs Beads. Blue fluorescence corresponds to the nuclei, green to the contrast agents and red to LAMP2, scale bar represents 10 µm. Transmission electron microscopy was carried out without post-staining and electron dense (dark) areas correspond to the iron-oxide based contrast agents.

All contrast agents displayed a perinuclear distribution (Figure 3). Molday ION and Feratrack showed a strong co-localisation with the lysosomal marker, LAMP-2, whereas for Bangs Beads, LAMP-2 staining was found mainly around the beads, without co-localisation. Transmission electron microscopy images corroborated these findings, with the contrast agents being found clustered in well-defined vesicles around the nucleus (Figure 3, right panels).

**Figure 4:**
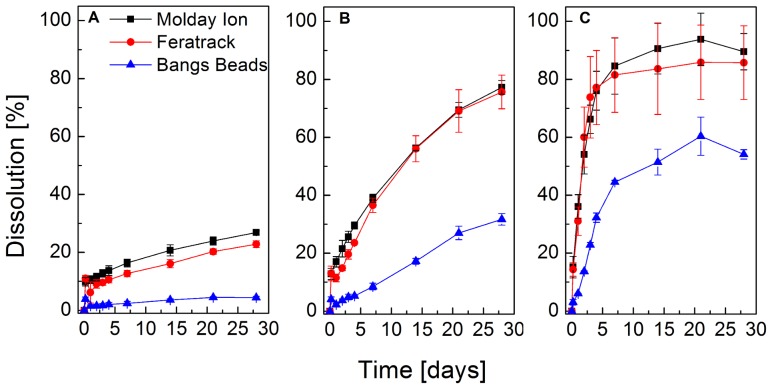
Dissolution of contrast agents in an *in vitro* model of lysosomal microenvironment. Contrast agents were allowed to digest in PBS containing 22(A) 7.2, (B) 5.5 or (C) 4.5. The relative dissolution is shown as a function of time for a period of 28 d. Data points represent mean ± SD from three independent experiments.

The relative dissolution of the contrast agents in citrate containing buffers at neutral (7.2) and endo-lysosomal (5.5–4.5) pH is shown in Figure 4. A pH dependent dissolution of the particles is observed in all conditions and at pH 7.2, only a small (<30%) dissolution that took place at a very slow rate is observed. Proportionally higher rates of dissolution were observed at pH 5.5 and 4.5, and Molday ION and Feratrack were found to fully dissolve within a period of 4 days when exposed to the lowest pH. Bangs Beads, on the other hand, exhibited a higher stability under all conditions displaying a maximum dissolution of 60% at pH 4.5.

### Labelling efficiency, intracellular retention and effect on MSC phenotype

**Figure 5:**
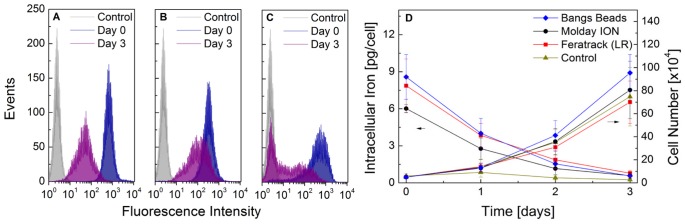
The labelling efficiency, intracellular iron content and cell proliferation as a function of time. Cells were labelled with the contrast agents for 24-well plates at a density of 5×10^4^ cells/well. Green fluorescence of unlabelled cells (control) or cells labelled with (A) Molday ION, (B) Feratrack and (C) Bangs Beads was evaluated following labelling (day 0) and at day 3. In (D), the intracellular iron (ferrozine assay, left ordinate) and cell proliferation (trypan blue exclusion, right ordinate) was measured at 24 h intervals. Data points correspond to the mean ± SD from three independent experiments.

Fluorescence measurements using flow cytometry reveal that when cells have been freshly labelled (day 0), the labelling efficiency is over 99% for Molday ION and Feratrack, whereas this figure is slightly lower for Bangs Beads, but still over 95% (Figure 5A–C). A more uniform labelling of cells with Molday ION and Feratrack, characterised by a histogram displaying narrower peaks, is observed when compared to Bangs Beads. After 3 days, a dilution of the number of particles per cell is observed, as identified by a marked decay in fluorescence intensity and a broadening of the histogram. For Bangs Beads, the uniformity of the labelling is broader from day 0 and after a growth period of 3 days, a bimodal distribution is observed suggesting a population of unlabelled cells whose green fluorescence intensity is equivalent to that of control (unlabelled) cells and another population containing intracellular particles (Figure 5C).

Intracellular retention of the contrast agents as measured by the ferrozine assay at 24 h intervals up to 3 days after labelling reveals that indeed all particles are diluted between daughter cells, resulting in an effectively lower intracellular concentration of iron as a function of growth time. The amount of iron per cell halved at approximately every 24 h, consistent with the cell's approximate doubling time (Figure 5D) and reached values close to that of control (unlabelled) cells after a period of 3 days.

**Figure 6:**
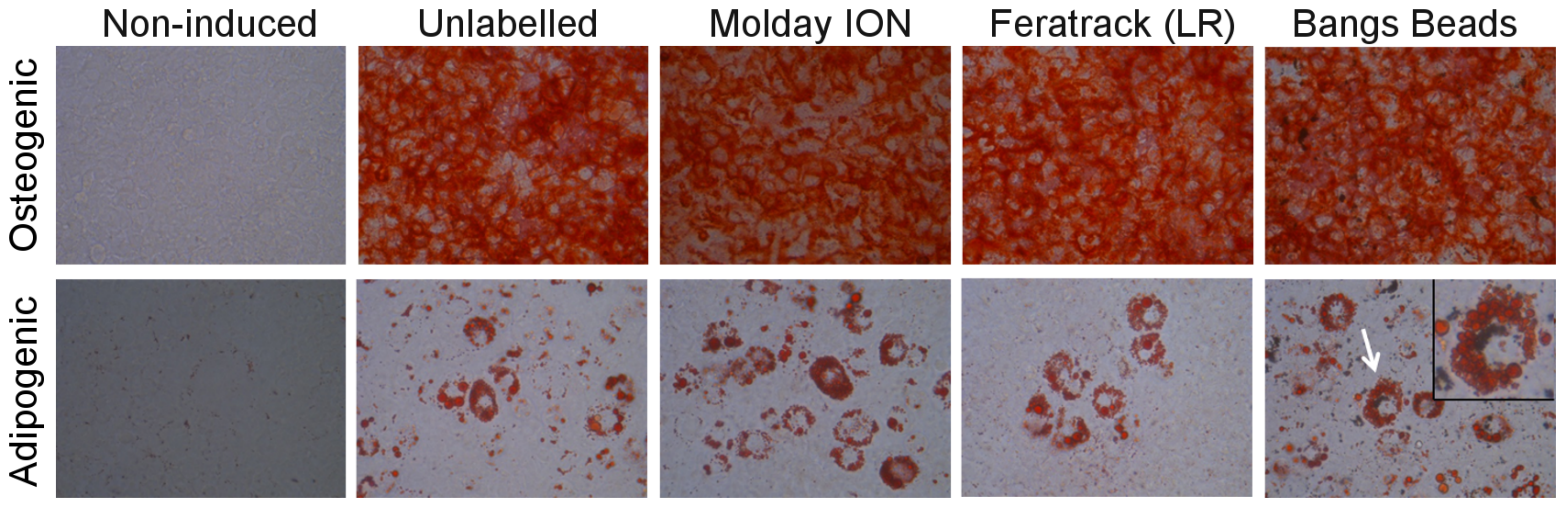
Differentiation of cells into osteocytes and adipocytes. Unlabelled (control) and labelled cells were induced to differentiate for 9 d with osteogenic or adipogenic media, after which cells were stained with Alizarin Red S to reveal calcium deposits or Oil Red O to reveal lipid droplets. Arrow indicates an adipocyte where the intracellular presence of labelling agent is clearly noticeable around the nucleus (zoomed view is shown in inset).

Trypan-blue exclusion assay conducted at 24 h intervals resulted in viability values >90% at every time point for all conditions. The negligible impact of the contrast agents on viability is reflected in the growth rate (Figure 5D) where labelled cells proliferated at rates similar to control cells, irrespective of the contrast agent used. In all cases, cells were capable of differentiating into osteocytes and adipocytes as efficiently as unlabelled cells as evidenced by a strong, positive staining for calcium deposits and intracellular lipid droplets, respectively (Figure 6). Because of their size and opacity, Bangs Beads were occasionally observed within adipocytes (Figure 6, arrows). Importantly, we found that if MSC are labelled with the contrast agents prior to inducing their differentiation, a strong signal could be detected with MRI for at least 9 days (the study end-point), which is likely due to the fact that these differentiated cells proliferate much more slowly than the undifferentiated MSC (Figure S3).

### Transverse Relaxivity *in vitro* and *in vivo*

**Figure 7:**
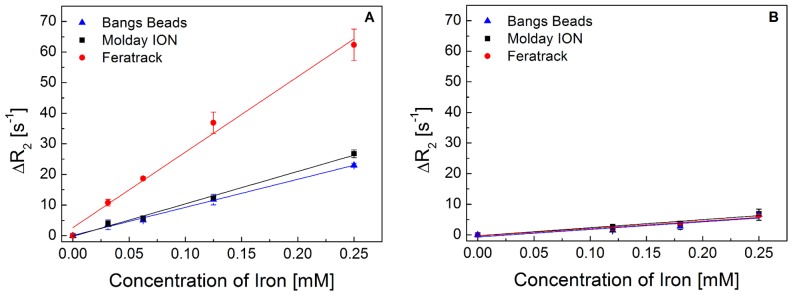
Relaxation rate (ΔR_2_) of contrast agents (A) in solution or (B) internalised in cells. Data points represent mean ± SD from three independent experiments and are displayed as a function of iron concentration. Lines represent the least square fit of the data points.

When measured in solution, the contrast agents displayed an expected concentration-dependent relaxation rate (Figure 7A) with Feratrack presenting the highest relaxivity at 247 mM^−1^s^−1^, nearly 2.5-fold higher than that measured for Molday ION and Bangs Beads at 106 and 92 mM^−1^s^−1^ respectively (Table 2). When the same measurements were carried out with labelled cells, however, a significant drop in relaxation rates was observed and all contrast agents displayed similar relaxivities at 26, 24 and 25 mM^−1^s^−1^ for Molday ION, Feratrack and Bangs Beads respectively.

**Table 2:** Transverse Relaxivity (r_2_) of contrast agents in solution or within cells. Values were obtained from the linear regression of the relaxation rate and are displayed as mean±SE from three independent measurements.

Contrast Agent	r_2_ in Solution (mM^−1^s^−1^)	r	r_2_ in Cells (mM^−1^s^−1^)	r
**Molday ION**	106±4	0.99	26±4	0.97
**Feratrack**	247±14	0.99	24±4	0.97
**Bangs Beads**	92±3	0.99	25±7	0.92

The correlation coefficient *r* is provided for each condition.

**Figure 8:**
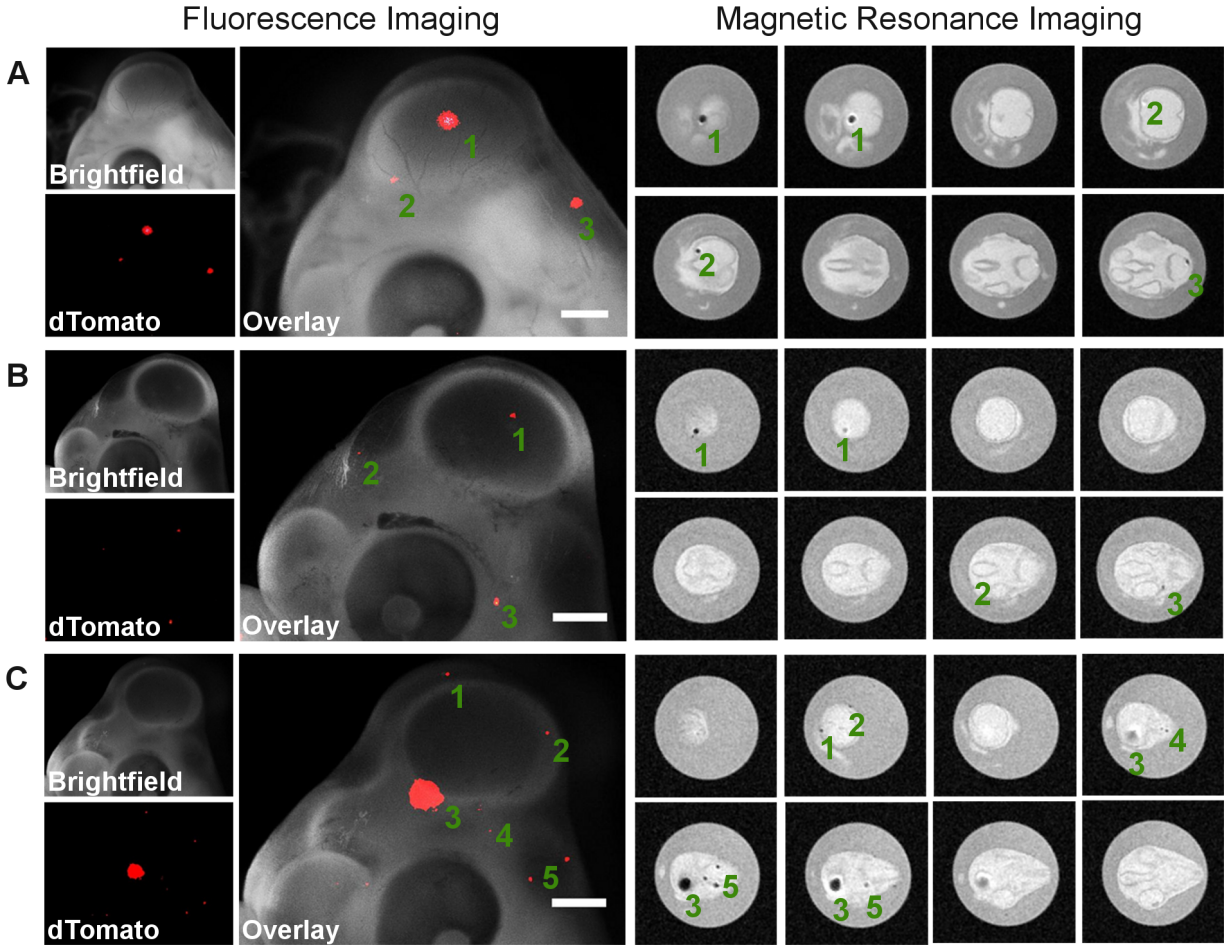
Fluorescence and magnetic resonance imaging of cells implanted into the brain of a chick embryo. Cells (5×10^4^) expressing dTomato were labelled with (A) Molday ION, (B) Feratrack or (C) Bangs Beads and implanted into the midbrain of chick embryos at embryonic day 3. At embryonic day 5 the embryos were harvested from their eggs, imaged with a fluorescence stereomicroscope and fixed prior to MR imaging using a T_2_-weighed RARE sequence. Scale bars represent 1 mm. Numbers in fluorescence images indicate the position of viable (dTomato expressing) cells. The corresponding numbers in the transverse MR sections show the T_2_ shortening effect of the labelling agent at the same anatomical positions.

Fluorescence imaging of chick embryos 48 h after implantation of 5×10^4^ labelled dTomato^+^ MSC into the midbrain revealed the presence of dTomato^+^ cells in several areas of the brain (Figure 8, left panel), confirming the cell's survival and integration in the host tissue. Although the cells were implanted at a single site, they spread to the fore-, mid- and hindbrain during the 48 h period between administration and analysis (from embryonic day 3 to 5). The fluorescence intensity, as well as the number of areas with positive cells, varied arbitrarily between samples, including chick embryos that were implanted with unlabelled dTomato^+^ cells (results not shown). These parameters (fluorescence intensity and number of positive areas) cannot thus be attributed to the presence and type of contrast agents within cells but are rather a reflection of biological variability.

MR imaging using a T_2_-weighed RARE sequence revealed the presence of areas of signal loss in several regions of the brain, which were found to correlate with the anatomical location of dTomato^+^ cells (Figure 8, right panel), confirming the suitability of all contrast agents, as well as of the labelling procedures, as a means for imaging *in vivo* using MRI. To test the likely effectiveness of the contrast agents for longer-term imaging, we developed a surrogate test system that involved culturing the labelled cells for 3 days prior to transplantation, in order to mimic an additional 3 days of growth *in vivo*. This approach was necessary because following the 5^th^ embryonic day, the increasing size of the chick embryo brain makes it difficult to visualise with fluorescence microscopy. We found that under these conditions, whilst no difference was observed in the intensity of the fluorescence signal between the 3 groups, the MR signal was noticeably stronger in embryos transplanted with MSC labelled with Bangs Beads (Figure S7).

## Discussion and Conclusions

### MR Contrast Agents & Cell Labelling

Iron oxide-based contrast agents are found in a variety of sizes and surface functionalities. As a rule, they consist of core-shell structures where the most common configurations are single core particles with a size below 100 nm or, multi-core particles consisting of an assembly of magnetic nanoparticles. Endorem, one of the former clinically approved contrast agents that proved popular for cell tracking, consisted of such multicore particles[13]. More recently, larger particles in the micrometer scale, consisting of a polystyrene matrix with several magnetic nanoparticle cores embedded within it, have also been used to label cells for MR imaging[14]. Here, we sought to compare the efficiency of these different particle configurations for cell tracking using mouse MSC. The therapeutic effect of MSC has been evaluated for a broad range of diseases demonstrating great clinical potential but major questions still remain, including their fate after administration[15]. These cells are therefore a relevant model for cell tracking. The nanoparticles chosen are representative of the configurations described above; Molday ION consists of a single core with a carboxydextran shell, whereas Feratrack is a multicore ensemble with a dextran shell. Bangs Beads are among the most popular micro-sized particles used for cell tracking and consist of a polystyrene matrix with several magnetic cores embedded within it.

It is well known that surface properties and the formation of a so-called “protein corona” [16], the layer of surface associated proteins that is formed in serum containing media, can play an important role in cell-nanomaterial interactions. Surface charge, in particular, seems to strongly influence cellular uptake, with positive charges often resulting in enhanced uptake by cells[17]. Of the particles examined here, Molday ION has a positive zeta potential whereas the carboxyl-modified polystyrene matrix of Bangs Beads leads to a negative surface charge. Feratrack, on the other hand, has a dextran shell which is usually reported as neutral[18] and thus in agreement with our results. Although these charges can be at least partially offset as a result of protein adsorption[17], [19], spontaneous uptake was only seen for the charged contrast agent Molday ION and Bangs Beads, which sediments in culture. Feratrack displayed negligible spontaneous uptake up to concentrations of up 100 µg[Fe]/ml and required the use of a loading reagent, disclosed by the manufacturer as lipid-based, to enhance cellular uptake. These characteristics of Feratrack are very similar to Endorem, which also consisted of multicore nanoparticles with a dextran shell[18] and likewise resulted in limited uptake by most cells requiring the use of transfection agents for efficient labelling[18], [20], [21].

Regarding the uptake of Bangs Beads, a common problem with particles of these dimensions is that they are not colloidally stable. Therefore, a strong sedimentation over the cells is observed that can be assumed to influence uptake. Furthermore, it is generally very difficult to wash away non-internalised particles once the labelling period is finished. Some investigators have mentioned success in separating labelled cells from extracellular aggregates using techniques such as density gradient centrifugation with the use of Ficoll-Paque[22]. In our hands, however, it was not possible to use this technique to obtain a clean and clear separation of cells from extracellular aggregates. In our experience, the concentration must be optimised to a level at which most particles will be taken up by cells during the labelling period, and therefore, precluding the need of extensive washes and the risk of carry-over of extracellular aggregates. With the cell line, cell density and labelling period used here, this was achieved with a concentration of 12.5 µg[Fe]/ml. Under these conditions, a strong sedimentation is seen in the initial hours of labelling but at the end of the labelling period, nearly all particles have been taken up by cells (Figure S1). This lack of colloidal stability and, as a consequence, uneven distribution of particles in solution is also likely to influence the fact that labelling with Bangs Beads is much less homogenous (as judged in terms of fluorescence intensity per cell in Figure 5C) than with other particles, which are colloidally stable.

With these data, we have thus identified conditions that provided the highest intracellular levels of iron, whilst at the same time, mitigated the presence of extracellular aggregates. Using these conditions, there was an expected small difference in intracellular iron between each of the contrast agents but which was not of statistical significance, allowing a direct comparison of the intracellular processing and imaging properties of these materials.

It is important to note that cellular uptake and response (*e.g.* toxicity) to different nanomaterials will vary depending on the origin and function of the cell being evaluated as a consequence of differences in surface proteins and sugars, as well as membrane composition; collectively, these characteristics have recently been coined "cell vision"[23]. For this reason, translating the labelling protocols described here to other cells might require adjustments and likewise, a systematic evaluation of intracellular iron content as a function of labelling conditions.

### Intracellular Processing and Toxicity

Once taken up by cells, TEM images indicated that particles were contained within well-defined vesicles, which have been identified as lysosomes by immunofluorescence staining for LAMP2. Co-localisation of LAMP2 and contrast agent fluorescence was limited to the rim of the Bangs Beads, an observation that can probably be attributed to the contrast agent's large size and the fact that LAMP2 is a lysosomal membrane protein. Because of this preferential localisation within lysosomes, which typically have a pH of 4.5–5.5, it is important to try to investigate the stability of the labelling agents within this organelle, given that SPION tend to degrade at low pH. Due to the difficulties with assessing SPION stability using live cells, Skotland *et al.* proposed in 1999[12] the use of acidic buffers with the presence of sodium citrate as a model of the lysosomal microenvironment. We found that the nanosized contrast agents underwent rapid dissolution at pH 5.5 and 4.5, which was comparable to that seen for similar products such as Clariscan[12] and Sinerem[24] but more rapid than has been reported for Endorem and Resovist[25]. Surprisingly, even the micro-sized contrast agents were found to partially dissolve at low pH 4.5, an unexpected observation given the non-biodegradable polystyrene matrix surrounding these particles. A closer examination of the particles revealed, however, that besides a strong polydispersity in size as seen in the TEM images in Figure 1, the core-shell structure of these contrast agents is not uniform. In fact, several single beads appear to have cores in direct contact with the surrounding environment (Figure S2) and are hence not completely encapsulated by the polystyrene shell. The iron oxide cores that are not completely encapsulated are thus likely to be in direct contact with the buffers, which likely explains the nearly 60% dissolution that is seen for this contrast agent at pH 4.5. With this observation accounted for, it is expected that the other 40% of those particles would not be digested *in vivo*. This non-biodegradability has an important impact in long-term cell tracking. On the one hand, it potentially allows the longitudinal tracking without the concern of progressively losing the contrast agent with time. On the other hand, it might exacerbate the issue of false positives; for instance, host cells that might engulf particles released from labelled cells that die following implantation. It is worth pointing out, however, that despite the fact that this assay has been widely used to estimate the intracellular stability of iron oxide-based contrast agents in the lysosomal microenvironment, it does have limitations and cannot completely mimic the conditions found in cells. For instance, we have been able to observe significant contrast in cells at least up to 9 days after labelling (Figure S3) suggesting that the dissolution does not occur as rapidly as observed under the conditions obtained using this lysosomal assay.

Apart from evaluating the intracellular processing within the lysosomes, it is also important to evaluate the effect of contrast agent dilution during cell division. One of the drawbacks when imaging labelled cells is that upon cell division (and potential migration within the host organism), the label is diluted, ultimately reducing the signal-to-noise when imaging. At the early stages of cell division, it is expected that the contrast agents are evenly distributed between daughter cells and it is possible to image that using live cell imaging (Figure S4). A comparison of fluorescence intensity between day 0 (freshly labelled cells) and day 3, however, reveals a significant difference between nano- and microsized particles. Whilst the former are evenly distributed between daughter cells during this period, resulting in a uniform peak of lower intensity at day 3 (Figure 5A,B), the latter displays an asymmetric distribution between daughter cells (Figure 5C). Because of the size of the particles, it is expected that when just one particle is present within a cell, such a situation would take place. This effect can be seen quite readily if the volume of the contrast agent is taken into account. A cell containing 8 pg of 8 nm nanoparticles contains a total of 8×10^6^ particles within its lysosomes, allowing their uniform distribution through several cell divisions. If the same particles are 1 µm, the same intracellular concentration of iron (8 pg) would correspond to only 4 particles allowing two cell divisions before asymmetric distribution between daughter cells. Live cell imaging of cells containing just one bead demonstrate that asymmetric distribution does take place *in vitro* (Figure S5). To confirm if the flow cytometry histogram obtained at day 3 corresponded to a bimodal distribution of labelled and unlabelled cells, we ran this sample through a magnetic-activated cell sorting (MACS) column, which allows the separation of the magnetically positive fraction. Flow cytometry analysis of this fraction (Figure S6) confirmed there are indeed two cell populations, one that did not contain any contrast agents (flow through), and a second population that retained the label (elution). For MR imaging, these observations are of relevance, for in the case of the microsized contrast agents, at least one of the daughter cells could potentially retain enough iron to display a T_2_-shortening effect, which would be lost in the case of nanosized particles, where a 50% reduction in signal intensity at every cell division would quickly render them undetectable. Shapiro *et al.* [26], have demonstrated this effect by inserting Bangs Beads into single cell mouse embryos and allowing them to develop up to embryonic day 7, and then using MRI to identify single cells within the organism that still contained the particles. Our own studies involving injection of cells in the brain of chick embryos 3 days after they had been labelled suggest an attenuated signal for the nanosized agents and a stronger signal for Bangs Beads (Figure S7).

Cellular toxicity of nanomaterials is a research area of great controversy[27]. It is generally accepted that there is a myriad of factors that can affect toxicity including the physicochemical properties of materials, intracellular content, exposure time, cell line, *etc.*, not to mention the wide range of assays which are available for evaluating different aspects ranging from cell proliferation to gene expression and DNA damage. Here, we have based our investigations on the evaluation of the hallmarks of stem cells: self-renewal and the capacity to give rise to specialised cells, and whether contrast agent labelling affected those properties. Proliferation of the MSC was unaffected by the labelling protocols used here and they were able to differentiate into osteocytes and adipocytes with the same propensity as unlabelled cells. In fact, in the case of Bangs Beads, it was possible to observe retention of several particles within single adipocytes, probably a consequence of cell arrest once they start to differentiate, preventing dilution of the label between daughter cells, and clearly suggesting that they do not affect their potential to differentiate into specialised cells. Beyond assays *in vitro*, studies using a chick embryo model show that cells with all contrast agents survive after implantation *in vivo* as evidenced by expression of dTomato 48 h after implantation into the animal.

### MR Imaging Properties

When the efficiency of contrast agents is considered, the standard property for assessing their efficacy is their relaxivity value[28]. The capacity of a T_2_ contrast agent to shorten relaxation times, thus leading to a hypointense signal in T_2_-weighed sequences, varies linearly with its concentration and the constant of proportionality corresponds to its relaxivity. Here, Feratrack was shown to display the highest relaxivity which was nearly 2.5-fold higher than Molday ION and Bangs Beads. It is generally acknowledged that magnetisation, particle and/or aggregate size and the volume fraction of contrast agents in the particle/aggregate are important factors in the design of contrast agents of high relaxivity[29]. Thus, although it is not possible to explain these relaxivity differences without a complete magnetic characterisation of the contrast agents, their morphology is likely to play an important role in these observations. Because Feratrack consists of multicore ensembles (aggregates) that can be considered as a large magnetised sphere[30] (a more efficient dephaser of proton relaxation), this would explain its high relaxation in respect to Molday ION, which consists of single particles. Relaxivity theories predict that particle size only increases relaxivity up to a certain extent with a maximum at a size range of 100 to 350 nm[29] after which relaxivity drops. This is the case for Bangs Beads, which consist of large particles of around 860 nm, and where the distance between the particles at a given iron concentration is so great that the diffusion of water in the magnetic field inhomogeneities is limited, resulting in a lower relaxivity.

A caveat of such measurements however, is that relaxivity is assessed under model conditions of colloidal suspensions of particles evenly distributed in aqueous solution. This is far removed from the intracellular situation seen here, where the contrast agents are clustered in the lysosomes. In fact, this clustering effect results in a similar configuration for all particles, where they all consist of aggregates enclosed within a cellular organelle with a size often larger than 500 nm. This configuration can limit diffusion on water just like the case for Bangs Beads in solution, reducing the relaxivity of all contrast agents when measured in cells. This effect can also be compounded by other intracellular effects. The diffusion coefficient of water, for example, is probably intrinsically lower in cells than in a pure solution and organelle membranes and intracellular compartments may also limit water diffusion and accessibility to the contrast agents[31]. This negative effect of intracellular confinement in transverse relaxivity has been previously shown for other iron oxide nanoparticles at low[32], [33] and high magnetic fields[34], and our results support such findings. An implication of these observations for T_2_-weighed cell tracking is that achieving high loads of intracellular contrast agents without affecting cell function might be of greater relevance than the development of contrast media with a high relaxivity.

To demonstrate the capacity of labelled cells to generate contrast *in vivo*, we have used a chick embryo model. We have opted to use this model in order to comply with the principles of the 3Rs. Furthermore, the absence of an immune system during the early embryonic stages allows xenogeneic cells to be administered [35] (here, murine stem cells), and also, the small size of the embryo during the first 5 days of development *in ovo* allows the use of non-specialised optical imaging to track cells. It was thus possible to evaluate the viability of cells using fluorescence imaging and then confirm their localisation and retention of contrast agents via MR imaging. We implanted the cells in the brain as it has a relatively large volume and longer T_2_-relaxation time when compared to other organs[36] and also because of its quick development, which we anticipated would result in a spread of cells and the possibility to evaluate the imaging properties of large and small cell clusters. In all cases, we could identify several regions in the brain which contained the administered cells. Because of the limitation of optical imaging, in particular signal attenuation due to tissue depth, it is not possible to directly correlate the signal intensity with the number of cells. Fluorescence imaging allowed us, however, to predict the anatomical localisation of the cells and confirm their viability *in vivo*. An excellent correlation was found between the position of the cells as evaluated optically and as imaged via MR, confirming the imaging properties of each of the contrast agents, and most importantly, their co-localisation with the administered cells even when those migrate to other parts of the brain. A significant increase in sensitivity was observed when using a Fast Low Angle Shot (FLASH) T_2_* sequence for imaging the embryos; however, that came at the expense of lower signal to noise for the rest of the tissue (Figure S8). The strong signal quench observed in the T_2_* sequence might also pose difficulties for detecting cell clusters in close proximity, as the hypointense areas can overlap. As the contrast in T_2_*-imaging is caused by the local magnetic field perturbation produced by the contrast agent, it extends well beyond the physical location of the particles. Thus it provides an amplification mechanism to detect low levels of labelled cells but does overestimate the volume occupied by labelled cells. The effect on T_2_, on the other hand, is better localised to the particles' physical location.

In summary, we evaluated the efficacy of nano- and microsized iron oxide-based contrast agents as a means to label and track cells. With proper adjustment in the labelling protocol, all contrast agents were successfully internalised within the lysosomes of murine MSC with intracellular levels in the range of 5–9 pg[Fe]/cell. All particles were retained within the cells *in vitro* and *in vivo* without presenting major cytotoxic effects. Although Feratrack offers a higher T_2_ relaxivity in solution, this is not reflected when internalised in cells where its imaging properties are equivalent to the other contrast agents. Thus, all contrast agents evaluated here are effective as a means to label and track cells with equivalent imaging properties once they are internalised in cells. Bangs Beads are less prone to dissolution and dilution between daughter cells in the long-term and can confer an advantage when cells that are proliferative and migratory are concerned.

## Supporting Information

Figure S1 **Representative images of MSC D1 during labelling.** Images were acquired during (5 h) and after the labelling period (24 h). There is noticeable sedimentation of particles at 5 h for cells labelled with Bangs Beads, resulting in a grainy image (detailed view in inset). After 24 h cells take up nearly all particles that have sedimented. For Feratrack, the labelling conditions (serum free medium, loading reagent) result in a small impact in cell morphology after 5 h (shrinkage) and proliferation after 24 h (reduced cell density). Data acquired with a Leica DM IL inverted microscope coupled to a DFC420C camera. Scale bar represents 100 µm. Prussian blue staining performed with an iron stain kit (Sigma) and imaged using bright field microscopy. For Bangs Beads no Prussian Blue staining is observed as the polystyrene matrix prevents the reaction of the staining reagent with the iron oxide cores.
(TIF)

Figure S2 **Transmission electron microscopy micrographs of Bangs Beads.** Particles which are not uniformly coated with the polystyrene shell are indicated with arrows.
(TIF)

Figure S3 **Magnetic resonance imaging of cell pellets before and after differentiation.** Images of cell pellets obtained using a T_2_-weighed RARE sequence. Cells were fixed and imaged directly after labelling or after a period of 9 days during which the cells were differentiated into adipocytes or osteocytes. Pellets obtained with cells differentiated into osteocytes are less uniform given the presence of a mineralised extracellular matrix.
(TIF)

Figure S4 **Live cell imaging of labelled MSC D1.** The symmetric distribution of Molday ION (top) and Bangs Beads (bottom) during mitosis is observed (overlay of phase contrast and fluorescence images acquired with a Zeiss LSM 510 Meta microscope). Feratrack is not included as it does not contain a fluorescent tag.
(TIF)

Figure S5 **Live cell imaging of MSC D1 10 d after labelling with Bangs Beads.** Once the contrast agent has been diluted between daughter cells, asymmetric distribution is observed during mitosis (overlay of phase contrast and fluorescence images acquired with a Zeiss LSM 510 Meta microscope).
(TIF)

Figure S6 **Magnetic retention of cells labelled with Bangs Beads.** Flow cytometry histogram (green fluorescence) of MSC D1 3 d after labelling with Bangs Beads and sorted with a Magnetic-Activated Cell Sorting (MACS) device. Flow thorough population displays no fluorescence whereas cells retained in the MACS column (elution) present a wide distribution of the contrast agent.
(TIF)

Figure S7 **Fluorescence and magnetic resonance imaging of cells implanted into the brain of a chick embryo.** Cells expressing a red fluorescent protein were labelled with (A) Molday ION, (B) Feratrack or (C) Bangs Beads and allowed to grow for a further 3 days to allow for the dilution of the contrast agents. After this period approximately 5×10^4^ cells were implanted into the midbrain of chick embryos at embryonic day 3. At embryonic day 5 the embryos were harvested from their eggs, imaged with a fluorescence stereomicroscope and fixed prior to MR imaging using a T_2_-weighed RARE sequence. Scale bars represent 1 mm. Numbers in fluorescence images indicate the position of viable (dTomato expressing) cells. The corresponding numbers in the transverse MR sections show the T_2_ shortening effect of the labelling agent at the same anatomical positions. In the case of the nanosized agents, although contrast is still obtained, the intensity is noticeable weaker than that obtained with freshly labelled cells.
(TIF)

Figure S8 Side-by-side comparison of samples scanned with a TurboRARE T_2_-weighed or FLASH T_2_* sequence. The slices contain hypointense regions corresponding to cells labelled with (A) Molday ION, (B) Feratrack and (C) Bangs Beads. An increase in the hypointense area is seen with the FLASH sequence. Conditions Turbo RARE T_2_-weighed: field of view 30×30 mm, matrix 256×256, slice thickness 1.0 mm, effective TE 33 ms, RARE factor 8, TR 2741.9 ms, averages 10, flip angle 135, scan time 14 min37 s, FLASH T_2_*: field of view 30×30 mm, matrix 256×256, slice thickness 1.0 mm, effective TE 15 ms, TR 450.8 ms, averages 4, pulse angle 30, scan time 7 min41 s. Average increase in hypointense area was 5.3-fold for Feratrack and Bangs Beads and 6.8-fold for Molday ION.
(TIF)
