# Correction: Assessing the Efficacy of Nano- and Micro-Sized Magnetic Particles as Contrast Agents for MRI Cell Tracking

**DOI:** 10.1371/journal.pone.0118037

**Published:** 2015-02-10

**Authors:** The PLOS ONE Staff

There is an error in the legend for Fig. 4 in the HTML version of the article. Please see the corrected Fig. 4 here.

**Figure 4:**
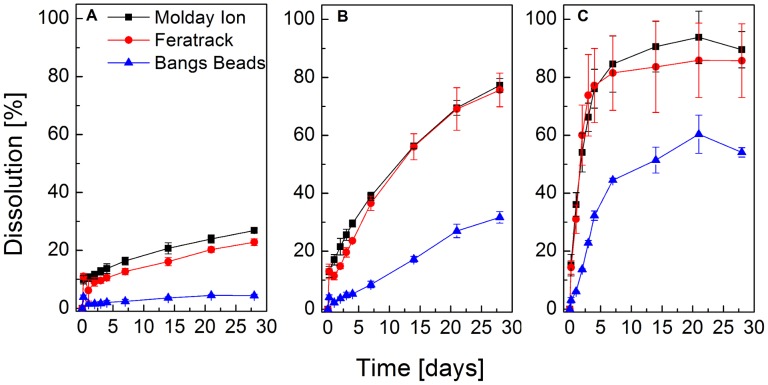
Dissolution of contrast agents in an in vitro model of lysosomal microenvironment. Contrast agents were allowed to digest in PBS containing 22 mM sodium citrate at pH (A) 7.2, (B) 5.5 or (C) 4.5. The relative dissolution is shown as a function of time for a period of 28 d. Data points represent mean +/- SD from three independent experiments.

There is an error in the legend for Fig. 5 in the HTML version of the article. Please see the corrected Fig. 5 here.

**Figure 5:**
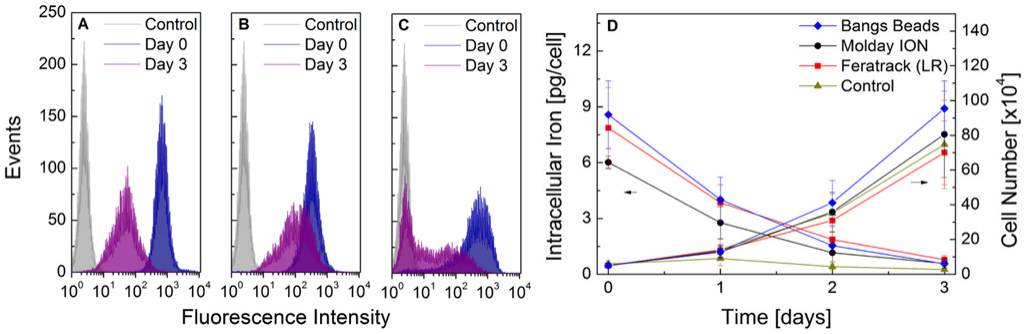
The labelling efficiency, intracellular iron content and cell proliferation as a function of time. Cells were labelled with the contrast agents for 24 h, trpysinised and seeded back in 24-well plates at a density of 5×104 cells/well. Green fluorescence of cells labelled with (A) Molday IONTM, (B) FeratrackTM and (C) Bangs Beads was evaluated following labelling (day 0) and at day 3. In (D), the intracellular iron (ferrozine assay, left ordinate) and cell proliferation (trypan blue exclusion, right ordinate) was measured at 24 h intervals. Data points correspond to the mean +/- SD from three independent experiments.
