# Supplementary figures and images for: Assessing the Efficacy of Nano- and Micro-Sized Magnetic Particles as Contrast Agents for MRI Cell Tracking
Source: PLoS One. 2014 Jun 24;9(6):e100259. doi: 10.1371/journal.pone.0100259 (PMC4069012; doi:10.1371/journal.pone.0100259)

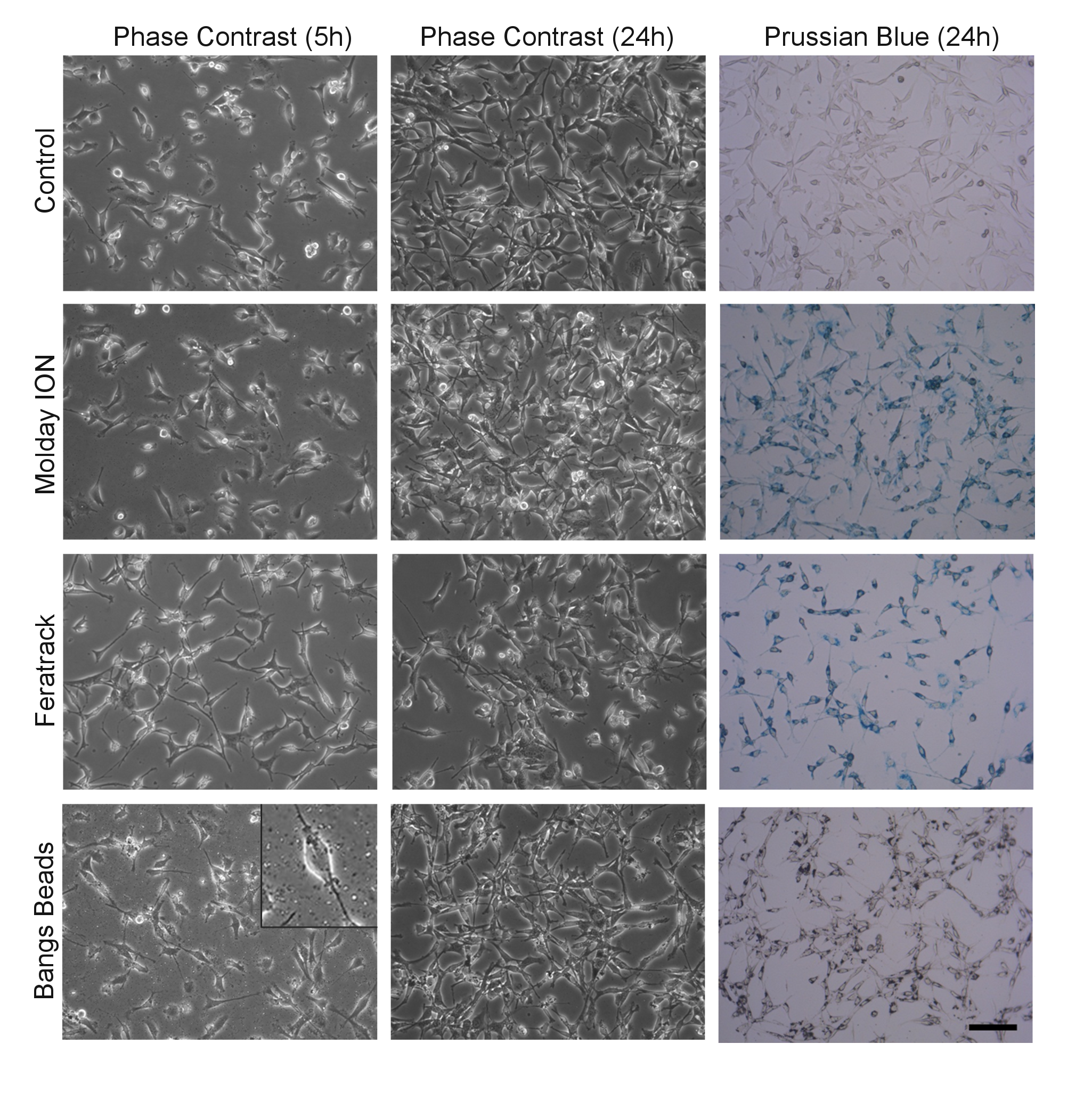

Supplement: Figure S1 — Representative images of MSC D1 during labelling. Images were acquired during (5 h) and after the labelling period (24 h). There is noticeable sedimentation of particles at 5 h for cells labelled with Bangs Beads, resulting in a grainy image (detailed view in inset). After 24 h cells take up nearly all particles that have sedimented. For Feratrack, the labelling conditions (serum free medium, loading reagent) result in a small impact in cell morphology after 5 h (shrinkage) and proliferation after 24 h (reduced cell density). Data acquired with a Leica DM IL inverted microscope coupled to a DFC420C camera. Scale bar represents 100 µm. Prussian blue staining performed with an iron stain kit (Sigma) and imaged using bright field microscopy. For Bangs Beads no Prussian Blue staining is observed as the polystyrene matrix prevents the reaction of the staining reagent with the iron oxide cores. (TIF) [file pone.0100259.s001.tif]

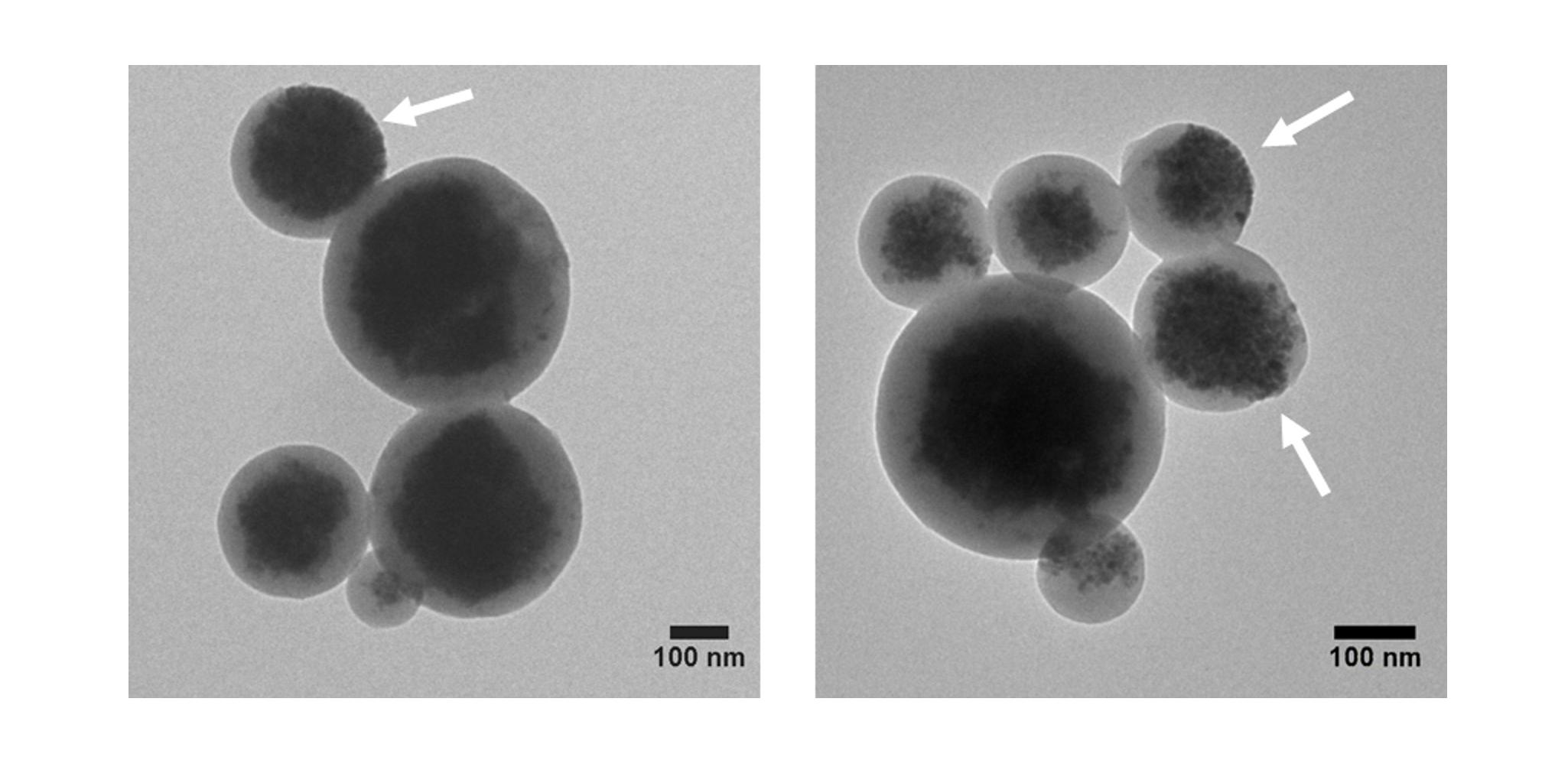

Supplement: Figure S2 — Transmission electron microscopy micrographs of Bangs Beads. Particles which are not uniformly coated with the polystyrene shell are indicated with arrows. (TIF) [file pone.0100259.s002.tif]

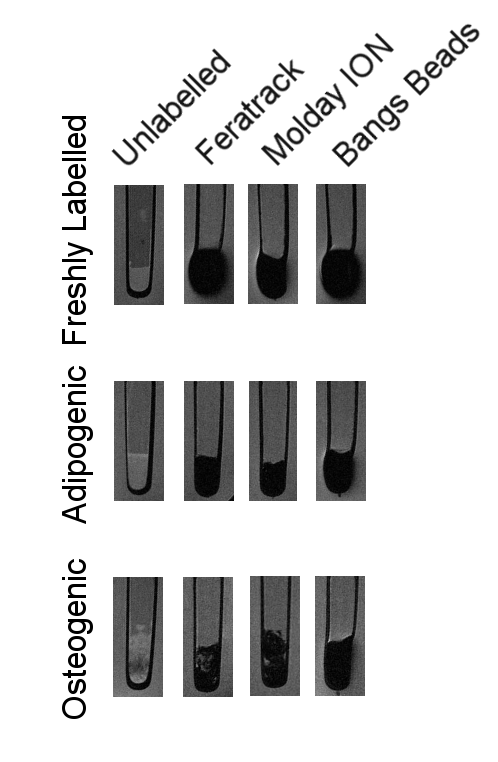

Supplement: Figure S3 — Magnetic resonance imaging of cell pellets before and after differentiation. Images of cell pellets obtained using a T2-weighed RARE sequence. Cells were fixed and imaged directly after labelling or after a period of 9 days during which the cells were differentiated into adipocytes or osteocytes. Pellets obtained with cells differentiated into osteocytes are less uniform given the presence of a mineralised extracellular matrix. (TIF) [file pone.0100259.s003.tif]

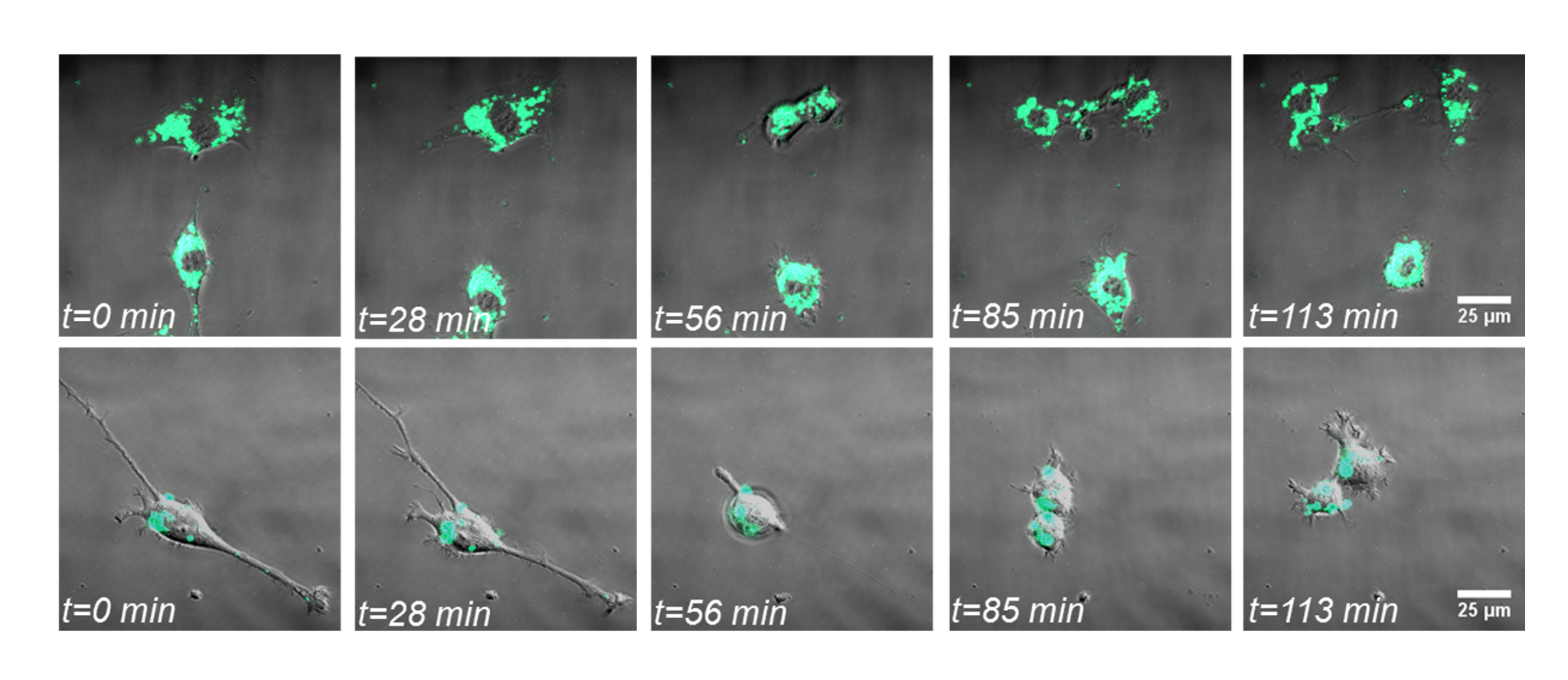

Supplement: Figure S4 — Live cell imaging of labelled MSC D1. The symmetric distribution of Molday ION (top) and Bangs Beads (bottom) during mitosis is observed (overlay of phase contrast and fluorescence images acquired with a Zeiss LSM 510 Meta microscope). Feratrack is not included as it does not contain a fluorescent tag. (TIF) [file pone.0100259.s004.tif]

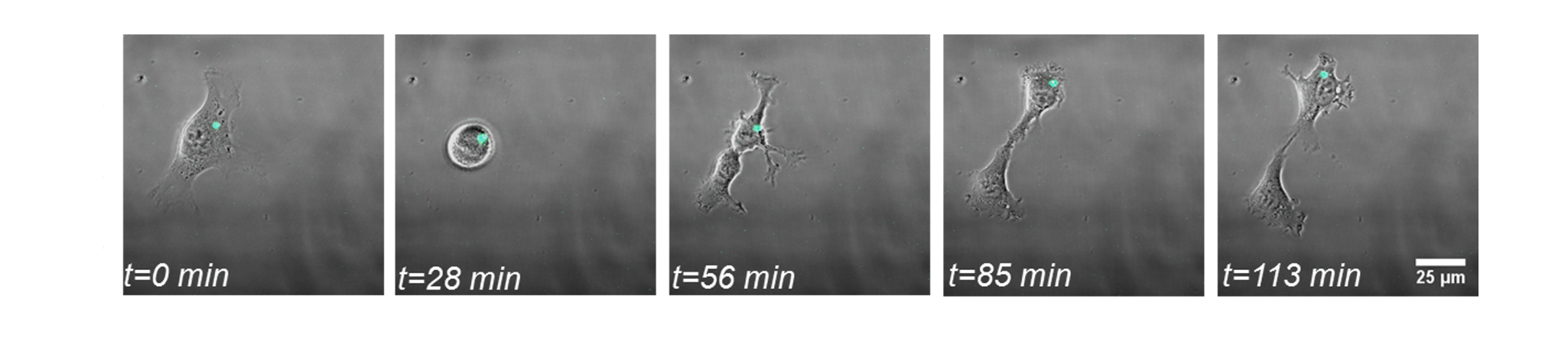

Supplement: Figure S5 — Live cell imaging of MSC D1 10 d after labelling with Bangs Beads. Once the contrast agent has been diluted between daughter cells, asymmetric distribution is observed during mitosis (overlay of phase contrast and fluorescence images acquired with a Zeiss LSM 510 Meta microscope). (TIF) [file pone.0100259.s005.tif]

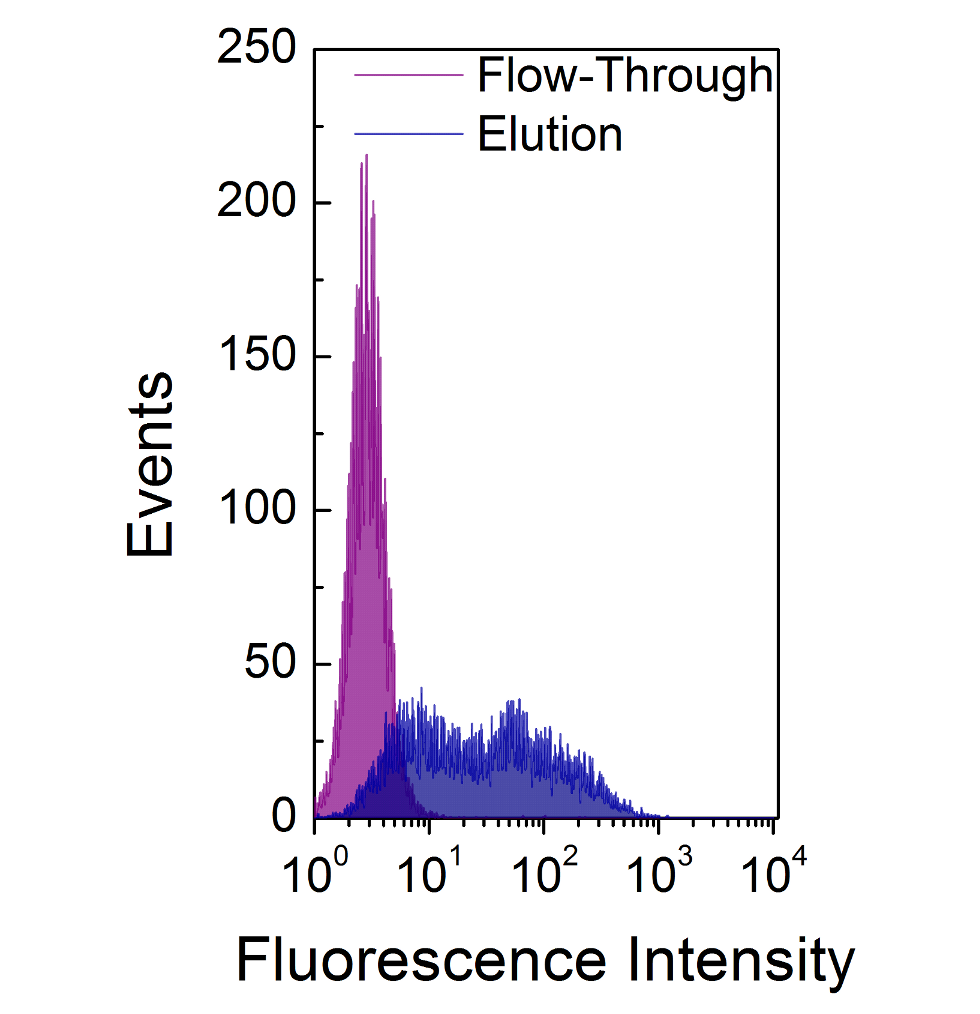

Supplement: Figure S6 — Magnetic retention of cells labelled with Bangs Beads. Flow cytometry histogram (green fluorescence) of MSC D1 3 d after labelling with Bangs Beads and sorted with a Magnetic-Activated Cell Sorting (MACS) device. Flow thorough population displays no fluorescence whereas cells retained in the MACS column (elution) present a wide distribution of the contrast agent. (TIF) [file pone.0100259.s006.tif]

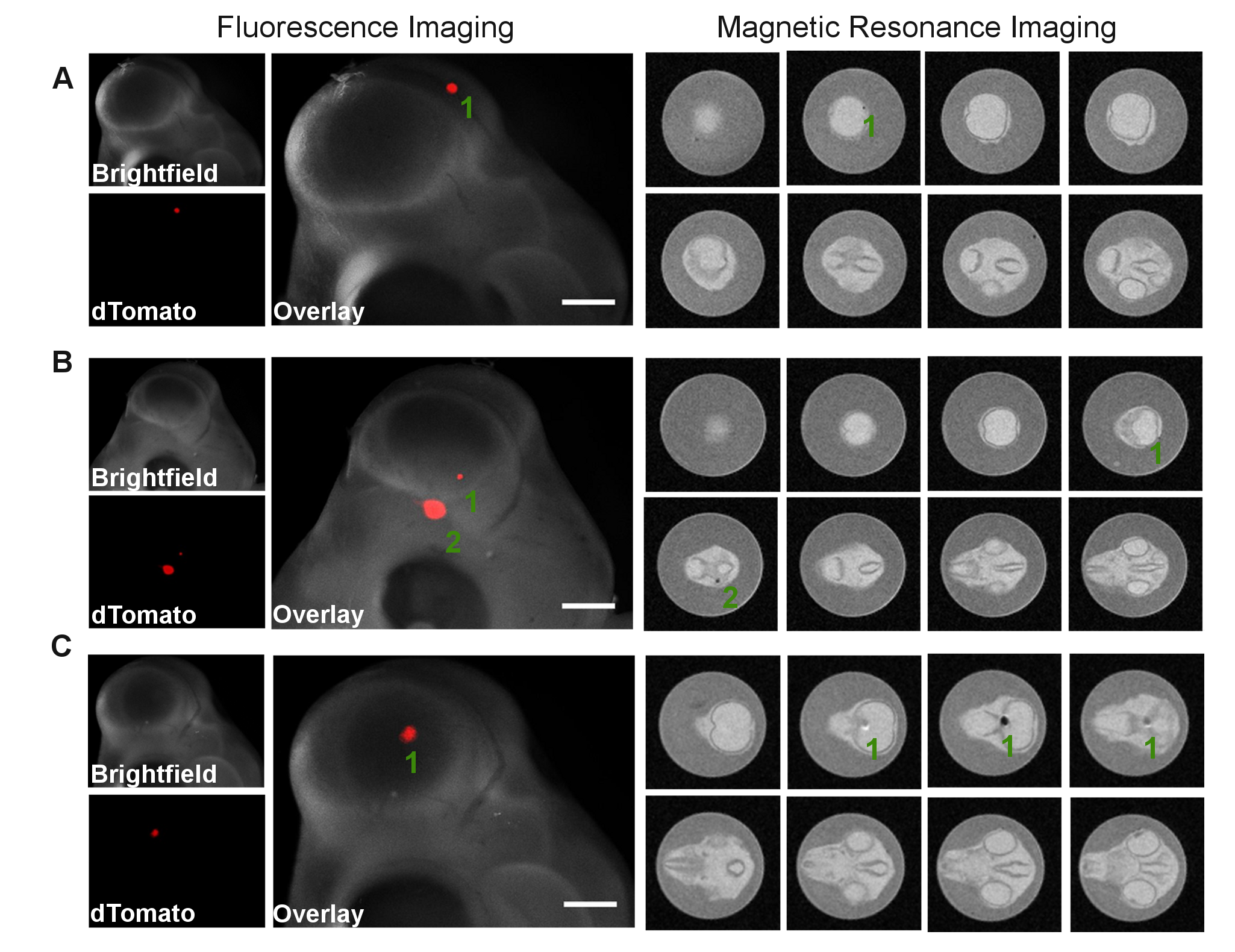

Supplement: Figure S7 — Fluorescence and magnetic resonance imaging of cells implanted into the brain of a chick embryo. Cells expressing a red fluorescent protein were labelled with (A) Molday ION, (B) Feratrack or (C) Bangs Beads and allowed to grow for a further 3 days to allow for the dilution of the contrast agents. After this period approximately 5×10^4^ cells were implanted into the midbrain of chick embryos at embryonic day 3. At embryonic day 5 the embryos were harvested from their eggs, imaged with a fluorescence stereomicroscope and fixed prior to MR imaging using a T2-weighed RARE sequence. Scale bars represent 1 mm. Numbers in fluorescence images indicate the position of viable (dTomato expressing) cells. The corresponding numbers in the transverse MR sections show the T2 shortening effect of the labelling agent at the same anatomical positions. In the case of the nanosized agents, although contrast is still obtained, the intensity is noticeable weaker than that obtained with freshly labelled cells. (TIF) [file pone.0100259.s007.tif]

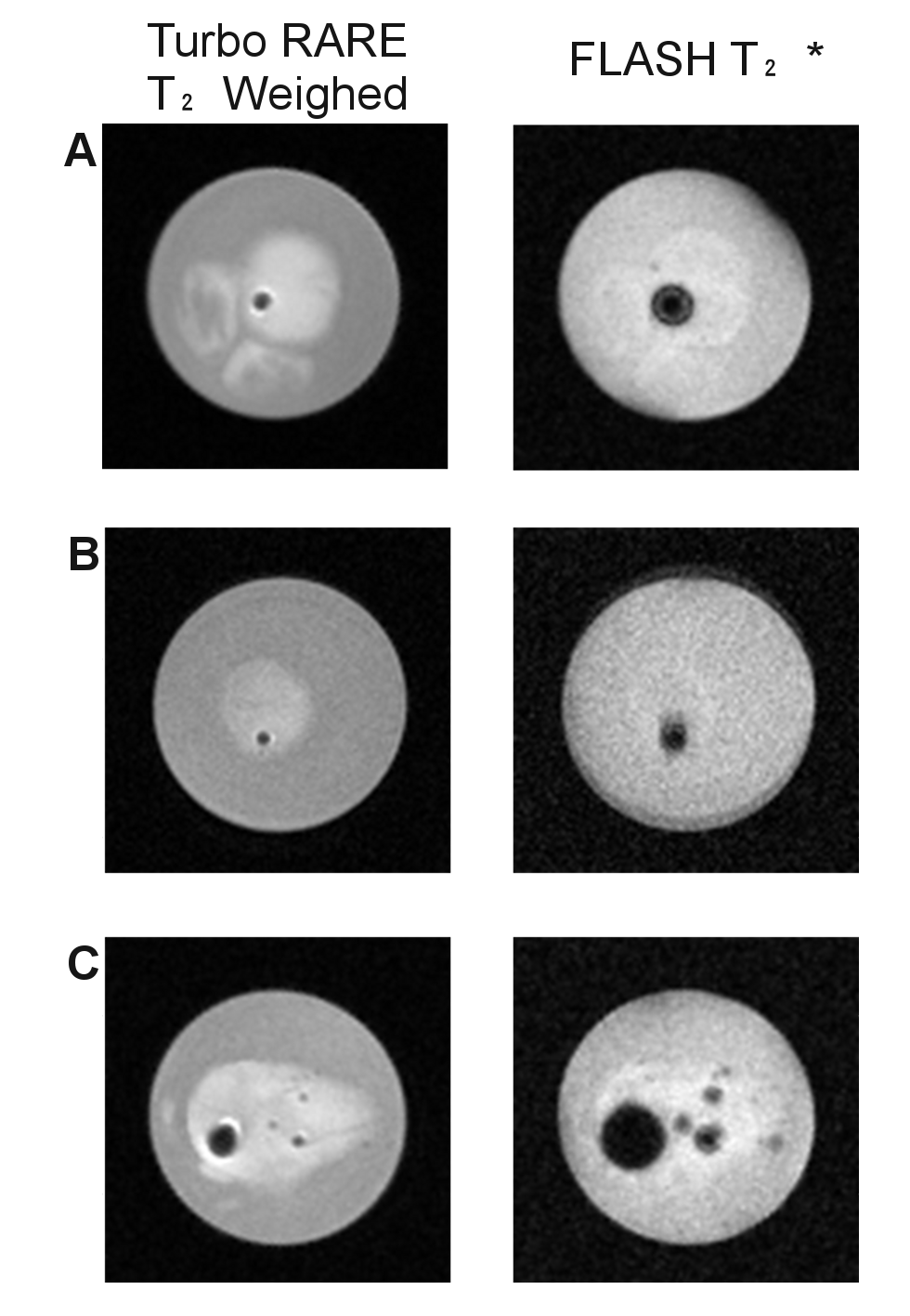

Supplement: Figure S8 — Side-by-side comparison of samples scanned with a TurboRARE T2-weighed or FLASH T2* sequence. The slices contain hypointense regions corresponding to cells labelled with (A) Molday ION, (B) Feratrack and (C) Bangs Beads. An increase in the hypointense area is seen with the FLASH sequence. Conditions Turbo RARE T2-weighed: field of view 30×30 mm, matrix 256×256, slice thickness 1.0 mm, effective TE 33 ms, RARE factor 8, TR 2741.9 ms, averages 10, flip angle 135, scan time 14 min37 s, FLASH T2*: field of view 30×30 mm, matrix 256×256, slice thickness 1.0 mm, effective TE 15 ms, TR 450.8 ms, averages 4, pulse angle 30, scan time 7 min41 s. Average increase in hypointense area was 5.3-fold for Feratrack and Bangs Beads and 6.8-fold for Molday ION. (TIF) [file pone.0100259.s008.tif]
